# A Case of Simple Melanoma of the Iris, with (Associated) Symptoms Simulating Simple Non-Inflammatory Glaucoma

**Published:** 1895-11

**Authors:** Joseph A. Mullen

**Affiliations:** 602 Main Street; Houston, Texas


### TEXAS MEDICAL JOURNAL.

ESTABLISHED JULY, 1885.

PUBLISHED MONTHLY.—SUBSCRIPTION $2.00 A YEAR.

VOL. XI. AUSTIN, NOVEMBER, 1895. No. 5.

For Texas Medical Journal.

### A CASE OF SIMPLE MELANOMA OF THE IRIS, WITH (ASSOCIATED) SYMPTOMS SIMULATING SIMPLE NON-INFLAMMATORY GLAUCOMA.

BY JOSEPH A. MULLEN, M. D., HOUSTON, TEXAS.

Read before the Houston District Medical Society, December 10th, 1894.

R. G., white, aged twenty-one years, occupation, clerk, has always enjoyed excellent health; there is no history of traumatism, either calvarial or ocular. General health good, emunctories normally active. No dyspnœa or œdema of eyelids or feet; never suffered from headaches; breathing full, regular and purely vesicular; respiration normal in strength and duration. Heart sounds clear and locally confined, steady in rythme and association. No symptoms of vertigo, either subjective or objective. No enlargements of the glandular organs or lymphatic system.

Urinary analysis showed the urine to be 1029, amber in color and acid in reaction, and on standing precipitated a large deposit of mucus, heavy in phosphates; no sugar or pigments.

The blood examination showed a reduction in the hæmaglobin from 13.44 per cent to 10.4 per cent.

The right eye is emmetropic and free from any pathological change or inflammatory condition. The field is normal in area and acuity; the retinal circulation is not disturbed; pupillary reaction prompt. No coloboma or excessive pigment deposit on the iris or choroid. The color of the iris is light blue; vision, $\frac{20}{20}$.

Left eye, on November 8th, 1894, $\frac{20}{80}$, and on the 22nd of same month it had fallen to $\frac{20}{200}$. The dark iris of the left eye produced a striking contrast, chromatically, with the light blue of the iris of the right, giving the impression on the right side of the face of a genial, pleasant disposition, and on the left, of a cold, calculating one. Pain is absent, and the loss of vision has been gradual. The reaction of iris to light and accommodation is entirely absent.

The melanosis was first noticed by the patient seven years ago and has gradually become more intensified; the chromatic asymmetry is marked.

The melanotic invasion of the iris is complete; the blood vessels are slightly enlarged, but not tortuous. The pupil is 3 m. m. in diameter and immobile.

There is no apparent increase in the muscular tissue of the iris, which is smooth and not undulated in any portion. The melanosis has partly saturated the sclera and its vessels; the latter being distinctly seen lying upon the steel-blue back ground.

The media are clear and emmetropic. The disc is cupped and partly surrounded by a scleral (?) ring. The depth of excavation is 1.04 m. m. The cup is probably pathological and indicates simple non-inflammatory glaucoma. The field of vision is limited to the temporal side, involving 3/8 of the temporal half of the field. There is no central scotoma or pulsations of the retinal vessels, when ocular pressure is exerted, nor halo of surrounding light. Intra-ocular tension is not increased. The temporal limitation of the field may possibly be due to the glaucomatous process, but if this is so it is, indeed, a rare anomaly. The anterior chamber is of normal depth, the right taken as a standard.

Melanomata are usually found either as simple or malignant growths, more rarely as the former, quite commonly as the latter, and are observed in those parts of the body in which there is normally a large amount of pigment, i. e., the choroid, iris, rete malpighii, lamina fusca and the pia mater. The melanotic sorcoma is more likely to occur in females than males, between the ages of twenty to forty years.

It may be of interest to dilate conservatively upon a few facts in pathology in connection with the subject of melanosis. Normal pigmentation is due to cell-action upon hæmaglobin (Green). May it not be that by some process, of which we are still ignorant, this physiological cell-action becomes functionally erratic, and as a result more than the requisite amount of hæmatoidin is produced and deposited in the elected tissues, and, as suggestively pointed out by Green, that probably hæmatoidin may have been merely altered by age, becoming darker by a heavier deposition of carbon, making it more insoluble and in the end constituting melanin. Then it would seem rational to infer that hæmaglobin retrogradedly becomes hæmatoidin, its granular or crystalline form depending partly upon the tissue in which it is localized, being ultimately transformed into melanin, not progressing as normally guided by a physiological instinct into bilirubin or urobilin, and passing out of the system as passively effete material.

An increased functional cellular activity necessarily implies a local acceleration of the circulation, and as a consequence more blood, the result of which there is an increase in cell action—this is corroborated by the fact that pigment deposits are bounteously supplied with blood. These deposits of vicarious hæmaglobin lie in the cells more frequently than the tissues between them, but their presence in either position is *per se* of little importance.

To a perversion of the influence or process, upon which its (pigment) formation depends, must be attributed the disturbance of the cellular elements which primarily causes normal pigmentation, secondarily melanosis, and unfortunately may produce malignancy, i. e., melanotic sorcoma.

It is only necessary in emphasizing this point to refer to a preceding simple melanoma, being the precursor of a consecutive melanotic sorcoma.

References to pigmentation, due to hæmatic metamorphosis, are implied as being referable only to localities in which pigment of quantities are found, i. e., choroid, iris, etc.

A fact worthy of consideration is the occasionally previous abnormal deposition of melanin before the appearance of a sorcoma.

Why is it in these unfortunately too frequent cases that this melanin is usually the destructive heralder of a consecutive melanotic sorcoma? Superficially it would seem that there is some connection between the causative element producing in these normally pigmented bodies a progressively slow and non-inflammatory accumulation of pigment upon which is occasionally precipitated a melanotic sorcoma.

May it not be, as already suggested, that the same present, unknown perversion, acting in a regular and consecutive manner, is capable of producing conditions in the tissue which, of themselves, are conducive to the development of malignancy? The result of the hæmatic examination would seem to suggest the inference that the perversion to which reference has been made is locally acting, and also that there are synchronous metamorphic changes of the hæmaglobin. This seems to be corroborated by the reduction in the coloring matter of the blood. In other words as the hæmaglobin passes through this erratic area (melanotic iris), its versatility is pathologically enhanced. The prognosis of melanoma iridis simplicis is vastly of more importance, both to the prolongation of life and the enjoyment of binocular vision, than is therapeusis, more particularly as the former depends upon the prophylactic measures pursued.

In reviewing the history of the case there are several factors which render the prognosis less favorable than otherwise; but, furthermore, there are still others which cast a shadow of uncertainty upon it. The unfavorable symptoms are somewhat rapid diminution in visual acuity, recent marked increase in the melanosis of the iris, the possibility of a pathological cupping of the optic disc (central vision preserved), absent pupillary reaction, rapid encroachment of the pigmentary deposit upon the sclera with increased vascularity of that membrane, and temporal limitation of the field.

The favorable factors are no hereditary history of malignant growths, no pain in the past or present, no evidence of a malignant body in the iris tissue, the long (seven years) benign history of the melanoma, no secondary sarcomatous deposit elsewhere, no increased intra-ocular tension, no retinal or choroidal changes, and the possibility of a physiological rather than a pathological disc cup, and the excellent health of the patient.

The line of treatment followed was purely prophylactic. Eserin sulphat was not instilled, as tension was normal.

Since writing the above, acute pain came on in the left eye, and subsided as quickly on the third day without further damage to V. With the view of probably modifying the suspected glaucomatous condition, and at the same time of obtaining some iris tissue for microscopical examination, and iridectomy, was suggested, but refused.

602 Main Street.

